# The Change of Teleost Skin Commensal Microbiota Is Associated With Skin Mucosal Transcriptomic Responses During Parasitic Infection by *Ichthyophthirius multifillis*

**DOI:** 10.3389/fimmu.2018.02972

**Published:** 2018-12-18

**Authors:** Xiaoting Zhang, Liguo Ding, Yongyao Yu, Weiguang Kong, Yaxing Yin, Zhenyu Huang, Xuezhen Zhang, Zhen Xu

**Affiliations:** ^1^Department of Aquatic Animal Medicine, College of Fisheries, Huazhong Agricultural University, Wuhan, China; ^2^Laboratory for Marine Biology and Biotechnology, Qingdao National Laboratory for Marine Science and Technology, Qingdao, China

**Keywords:** mucosal immunity, skin, transcriptomic, microbiota, *Ichthyophthirius multifiliis*, rainbow trout (*Oncorhynchus mykiss*)

## Abstract

Teleost skin serves as the first line of defense against invading pathogens, and contain a skin-associated lymphoid tissue (SALT) that elicit gut-like immune responses against antigen stimulation. Moreover, exposed to the water environment and the pathogens therein, teleost skin is also known to be colonized by diverse microbial communities. However, little is known about the interactions between microbiota and the teleost skin mucosal immune system, especially dynamic changes about the interactions under pathogen infection. We hypothesized that dramatic changes of microbial communities and strong mucosal immune response would be present in the skin of aquatic vertebrate under parasite infection. To confirm this hypothesis, we construct an infected model with rainbow trout (*Oncorhynchus mykiss*), which was experimentally challenged by *Ichthyophthirius multifiliis* (Ich). H & E staining of trout skin indicates the successful invasion of Ich and shows the morphological changes caused by Ich infection. Critically, increased mRNA expression levels of immune-related genes were detected in trout skin from experimental groups using qRT-PCR, which were further studied by RNA-Seq analysis. Here, through transcriptomics, we detected that complement factors, pro-inflammatory cytokines, and antimicrobial genes were strikingly induced in the skin of infected fish. Moreover, high alpha diversity values of microbiota in trout skin from the experimental groups were discovered. Interestingly, we found that Ich infection led to a decreased abundance of skin commensals and increased colonization of opportunistic bacteria through 16S rRNA pyrosequencing, which were mainly characterized by lose of *Proteobacteria* and increased intensity of *Flavobacteriaceae*. To our knowledge, our results suggest for the first time that parasitic infection could inhibit symbionts and offer opportunities for other pathogens' secondary infection in teleost skin.

## Highlights

- Skin mucosal immunity interacts with the commensal microbiota that adhere to the epidermis.
- Parasitic infection leads to commensal microbial dysbiosis in trout skin.
- Successful invasion of Ich benefits the colonization of pathogens in teleost skin.
- Skin mucosal immunity can be activated by both parasitic infection and the subsequent infection caused by pathogens.

## Introduction

The skin of vertebrates serve as the first line of defense against pathogens' invading and harbor millions of microorganisms that form a very ancient and successful symbiosis between prokaryotes and metazoans. In all vertebrates, the skin shares a similar structure including two main layers (epidermis and dermis). In contrast to mammalian skin, teleost skin has been considered as mucosal surface that harbors abundant mucus-producing cells, lacks keratinization, and possesses living epithelial cells that make direct contact with the water medium (1, 2). Importantly, besides as one of important mucosal tissue, teleost skin has been discovered to contain a skin-associated lymphoid tissue (SALT) and elicit gut-like immune responses (3). Moreover, teleost skin is also known to be colonized by diverse microbial communities (4). The presence of bacterial communities in teleost skin also suggests a tight cross-talk between the microbiota and mucosal immune system. Although many studies have revealed the interaction between microbial communities and the host mucosal immune system of teleost fish (5–9), information regarding that in the skin remains limited (10).

As previously stated, the harmonious relationship between host and microbiota can be disrupted by pathogen infection, which lead to dysbiosis (11, 12). However, the distinction between a symbiont and pathogen is often blurry. Generally, symbionts are defined as microorganisms that induce anti-inflammatory cytokine expression in mucosal tissues, whereas pathogens induce pro-inflammatory responses (13, 14). Differently, in some cases, commensal will eventually trigger pro-inflammatory responses if present in high enough numbers (15). In addition, previous studies have shown that during the course of an infection, the relationships between the microbiota and the host's immune system are vulnerable to changes that render commensals into opportunists or pathogens (16), and largely impact the outcomes of infections (6). Thus, it's suggested that commensal microbiota play a fundamental role in mediating host–parasite interactions (9, 17, 18). Importantly, a number of studies have shed light on the changes in immune genes or bacterial communities at teleost mucosal sites after infection with different pathogens (16, 19, 20). Moreover, only few studies have taken advantage of the whole-transcriptome approach to investigate changes in immune gene expression in fish intestine, gills, skin, and olfactory organ (21–28). However, by far, almost no research has been performed to detect interactions between the microbiota and the SALT of teleost during parasitic infections.

Here, using both the transcriptome and microbiome, our study examined the relationships between mucosal immunity and microbiota on the skin surfaces in teleost challenged with a parasite, *Ichthyophthirius multifiliis*, which poses the threat of serious economic loss to the worldwide trout farming industry (29). Our findings show that Ich successfully invading skin mucosa based on paraffin sections of skin stained with H & E. Moreover, we discover that the mucosal layer was destroyed by Ich, leading to heavy histopathological damage and microbial dysbiosis in trout skin. Importantly, significantly increased expression levels of numerous immune-related genes were discovered in trout skin after infection. In addition, the microbial dysbiosis in the skin of rainbow trout suggests that Ich has important inhibitory effects on commensals. Furthermore, our results show that skin mucosal immunity can be activated by both parasitic infection and the subsequent infection caused by opportunistic pathogens and pathogens. These results firstly demonstrate that the skin mucosal immune responses are associated with changed commensal microbiota after parasitic infection in teleost fish.

## Materials and Methods

### Experimental Fish

Rainbow trout (10 ± 1 g) used in this study were obtained from fish farm in Shiyan (Hubei province, China), and maintained in aquarium tanks using a water recirculation system involving thermostatic temperature control and extensive biofiltration. Fish were acclimatized for at least 2 weeks at 15°C and fed with commercial trout pellets at a rate of 0.5–1% biomass twice a day (9:00 a.m. and 5:00 p.m.), and feeding was terminated 48 h prior to sacrifice. Animal procedures were approved by the Animal Experiment Committee of Huazhong Agricultural University and carried out according to the relative guidelines.

### Ich Parasite Isolation and Infection

The methods used for parasite isolation and infection were described previously by Xu et al with slight modification (30). Briefly, heavily infected rainbow trout were anesthetized with overdose of MS-222 and placed in a container with water to allow trophonts and tomonts exit the host. The fish were taken out of the water after 4 h while the trophonts and tomonts were left in the water at 15°C for about 24 h to let tomocyst formation and subsequent theront release.

For parasite infection, fish were exposed to an optimal single dose of ~5,000 theronts per fish added directly into the aquarium. Then tissue samples were taken after 12, 24, 48, 72 h, 7, 21, and 28 d (infected fish). Every experiment was performed at least three times. Control fish (mock infected) were maintained in a similar tank but without parasite. Fish were starved the days when sampling was conducted.

### Sample Collection

For sampling, rainbow trout were anesthetized with MS-222 and tissues were collected at the indicated time points after infection. For histology and pathology study, a piece of the dissected skin (~0.25 cm^2^) under dorsal fin was clipped and immediately fixed in 4% (v/v) neutral buffered paraformaldehyde for at least 24 h. For RNA extraction and quantitative real-time PCR (qRT-PCR), tissue samples including skin, spleen and head kidney for all time points after infection were collected in sterile micro-centrifuge tubes. For 16S rRNA sequencing, skin samples from 24 h and 7 d post-infected fish and control fish were collected in sterile freezing tubes. All of these tissues collected for RNA or DNA analyses were immediately froze with liquid nitrogen and stored at −80 °C for further study.

### Histology, Light Microscopy, and Immunofluorescence Studies

After dissected and fixed in 4% neutral buffered formalin at 4°C, the trout skin tissues were then dehydrated in a graded ethanol series, cleaned in xylene, embedded in paraffin, and cut in sections of 5 μm in duplicate with a rotary microtome (MICROM International GmbH, Germany). A copy of sections were stained with classical hematoxylin and eosin (H & E), while another portions were conventional stained with alcian blue (A & B). All the stained sections were examined under light microscope (Olympus, Japan). Quantification was performed by three different researchers.

Immunofluorescence staining was performed on paraffin sections obtained from the skin tissue of rainbow trout. For the detection of Ich parasite, paraffin sections were incubated overnight at 4°C with rabbit anti-Ich (0.2 μg ml^−1^) or its isotype control (rabbit IgG1 isotype; 0.2 μg ml^−1^). After being washed three times with PBS (pH 7.2), samples were incubated for 2 h at 20°C with secondary antibody Alexa Flour® 488-AffiniPure goat anti-rabbit IgG (H + L; 115-545-003; 2.5 mg ml^−1^, Jackson Scientific, Germany). Before mounting, all samples were stained with DAPI (4′, 6-diamidino-2-phenylindole; 1 mg ml^−1^; Invitrogen). Images were acquired and analyzed using Olympus BX53 fluorescence microscope (Olympus) and the iVision-Mac scientific imaging processing software (Olympus).

### RNA Isolation and Quantitative Real-Time PCR (qRT-PCR) Analysis

All the samples for quantitative real-time PCR (qRT-PCR) analyses were homogenized in 1 ml TRIZol (Invitrogen Life Technologies) using steel beads shaking (60 HZ for 1 min) and then subjected to total RNA extraction according to the manufacturer's instructions. The concentrations of the extracted RNA were quantified with a NanoDropND-1000 spectrophotometer (Thermo Scientific) and the integrity of the RNA was determined by agarose gel electrophoresis. To normalize gene expression levels for each sample, equivalent amounts of the total RNA (1 μg) were incubated with RNase-free DNase I to eliminate contaminated genomic DNA. Then reverse transcription was performed using SuperScript first-strand synthesis system (Abm, Canada) in a 20 μl reaction volume. The resultant cDNA was stored at −20°C.

The relative expressions of immune-related genes were determined by qRT-PCR with each specific primer (Table 1). The qRT-PCR were performed on a 7500 Real-time PCR system (Applied Biosystems, USA) using the EvaGreen 2 × qPCR Master mix (Abm, Canada). The total volume of qRT-PCR amplification system were 10 μl, containing 5 μl Master mix, 1 μl diluted cDNA (300 ng), 0.25 μl of each gene specific primer (10 μM), and 3.5 μl nuclease-free water. The internal control gene elongation factor 1α (EF1α) was employed as reference gene. To detect the relative abundance of Ich and *Flavobacterium columnare* (*F. columnare*), specie-specific 18S rRNA and 16S rRNA primers were used and shown in Table 1, respectively. All samples were performed as following conditions: 95 °C for 30 s, followed by 40 cycles at 95 °C for 1 s and at 60 °C for 10 s. A dissociation protocol was carried out after thermos cycling to determine the specific-sized single application. Relative mRNA abundances were calculated using the 2^−ΔΔCt^ method and normalized to EF1α. The relative expression levels of immune-related genes were shown as 2^−ΔΔCt^ while the relative abundance of Ich and *F. columnare* were shown as –ΔΔCt without further calculated to 2^−ΔΔCt^. All data were expressed as mean ± standard error estimate (s. e. m.). Student's *t*-test was conducted using GraphPad 5.0. The results were obtained from three independent experiments and each was performed in triplicate.

**Table 1:** Gene-specific primers used for quantitative real-time PCR in this study.

**Gene**	**Primer**	**GenBank accession no**.	**Tm (^**°**^C)**	**Amplicon length (bp)**	**Primer Sequence (5**^**′**^**-3**^**′**^)	
					**Forward primer**	**Reverse primer**
**IMMUNOGLOBULINS AND THEIR RECEPTOR**						
IgT	IgT-F/IgT-R	AY870264	58		CAGACAACAGCACCTCACCTA	GAGTCAATAAGAAGACACAACGA
IgM	IgM-F/IgM-R	S63348.1	58		AAGAAAGCCTACAAGAGGGAGA	CGTCAACAAGCCAAGCCACTA
IgD	IgD-F/IgD-R	AY748802.1	58	138	CAGGAGGAAAGTTCGGCATCA	CCTCAAGGAGCTCTGGTTTGGA
pIgR	pIgR-F/pIgR-R	FJ940682.1	58	145	GTACAGCAGGTGTTCACAGTAAC	CCACAGACAGACCTTGGATAAC
**COMPLEMENT AND PATHWAY RELATED GENES**						
Complement 3	C3-F/C3-R	XM_021561577.1	58	213	CCTCACAACAAGAGTGCACATC	CCAAGTGGGCAAACTCATCTCC
Complement 1q-2	C1-F/C1-R	XM_021624859.1	58	176	TGAACAACCTGAACACCCC	CAGCCTATTAGCCTGTAACTCC
Complement 7-1	C7-F/C7-R	NM_001124618.1	58	218	TATCTTCACTGCCACGGTC	TAGCCTGTAACTCCACATAGAC
**CYTOKINES**						
interleukin 2	IL2-F/IL2-R	AM422779.1	58	125	TGTCTACAAGGAAACCCAA	GCTGCAACAATGCAACTAT
interleukin 11	IL11-F/IL11-R	NM_001124382.1	58	191	CAGAGCGTCAAGGAAACAC	GCTCCTGGGAAGACTGTAA
interleukin 22	IL22-F/IL22-R	AM 748537	58	101	GCAGGACAAGCATCATCCTAA	CATTGTAGTGTTTAATAGCACAGC
interferon α	IFNα-F/IFNα-R	XM_021578986.1	58	180	CAGAGCCTCAGGAAGAACT	CAAGGGGTAGAAGAGCATA
IRF 1-2	IRF1-F/IRF1-R	NP_001239293.1	58	173	CGAGACTACACCAGACCCTA	TTGCTTTTGACCTCTTGTTATT
TNF α	TNFα-F/TNFα-R	XM_021576327.1	58	204	GTATGCGATGACACCTGAA	GCCCCATTAGAGTGCCTTA
CCL 19	CCL19-F/CCL19-R	AIN40032.1	58	139	GTTTCCCTCGCCACTTCAA	GCCACCCACTTGCTCTTTG
***ICHTHYOPHTHIRIUS MULTIFILIIS***						
18S rRNA	Ic18-F/Ic18-R		60		AGTGACAAGAAATAGCAAGCCAGGAG	ACCCAGCTAAATAGGCAGAAGTTCAA
***FLAVOBACTERIUM COLUMNARE***						
16S rRNA	Fc16-F/Fc16-R	EU395796.1	60	146	GAGTGGCTAAGCGAAAGTGAT	ACCTGACACCTCACGGCAC
**REFERENCE GENE**						
EF-1α	EF1-F/EF1-R	XM_021571866.1	58		CAACGATATCCGTCGTGGCA	ACAGCGAAACGACCAAGAGG
β-actin	BA-F/BA-R	AB196465	60	225	CCGCGACCTCACAGACTACC	GTGCCCATCTCCTGCTCAAA
**OTHERS**						
cathelicidin 1	CATH1-F/CATH1-R	NM_001124480.1	58	152	CTGGAGGCAAGCAACAAC	CCCCCAAGACGAGAGACA
COL1α	COL1α-F/COL1α-R	NM_001124206.1	58	190	ATGTGCGACGAAGTGATTTG	CGTTACCTGGAACACCCTC
m-csfr	CSFR-F/CSFR-R	NM_001124739.1	58	120	CCCGCCTGTCACCCAATCT	CGTCCCACCAATGCTTCT
C-type lectin 4E	CTL4-F/CTL4-R	XM_021562202.1	58	137	GCAGCCACCTTACCATC	CACCCATCTCCAATCCC
DnajC3	DC3-F/DC3-R	XM_021597283.1	58	166	TATTGAAACGTGTGTGTGGG	AGGTGGTAGTAGATGGTGCTG
DnajB9	DB9-F/DB9-R	XM_021577464.1	58	234	CCGCTGTTGTTAGTGCTTT	CGTCAGGACTCTTGTTTCG
ENO	ENO-F/ENO-R	XM_021578347.1	58	214	TCAAGTCACCTGACGACCCT	ACAGCCTGCTGGATACGC
FHL1	FHL1-F/FHL1-R	XM_021618853.1	58	212	CGGCTCTGAGAATGTGG	ATGGCTGCTCCTGGTAG
GAPDH	GAPDH-F/GAPDH-R	NM_001124209.1	58	100	GACACCCACTCCTCCATCTT	TGCTGTAGCCAAACTCATT
LAMβ3	LAMβ3-F/LAMβ3-R	XM_021570344.1	58	211	TCAGGGTTCGGAGGTCGTA	CACAGTCGGGAAAGGAGTAA
LDH	LDH-F/LDH-R	XM_021605689.1	58	105	ACTCCAGCGTGCCCGTAT	CGTGCTTCCAGTCCTCCTT
PFKA	PFKA-F/PFKA-R	XM_021609920.1	58	103	CGACACCCGTTCCACAAT	AGCCATCACAGCCTCCAC

*The primers of IgT, IgM, and EF1α were obtained from the previous study of Tacchi et al. (24)*.

### RNA-Seq Library Construction, Sequencing, and Analyses

The RNA samples of trout skin were sent to Novogene Bioinformatics Technology Co. Ltd. (Beijing, China), where the quality and quantity of total RNA were determined by a Nano Photometer spectrophotometer (IMPLEN, CA, USA), a Qubit 2.0 Fluorometer (Life Technologies, CA, USA), and a Agilent 2100 bioanalyzer (Agilent Technologies, CA, USA). Then the mRNA were purified by poly-T oligo-attached magnetic beads and fragmented by divalent cations in NEB Fragmentation Buffer. Random hexamer primers and M-MLV Reverse Transcriptase (RNase H) were used for first strand cDNA synthesis. Second strand cDNA synthesis was subsequently performed using DNA polymerase I and RNase H. These double-stranded cDNA fragments were end-repaired by adding a single “A” base and ligation of adapters. The adaptor modified fragments were selected by AMPure XP beads and amplified to create the final cDNA library. DGE sequencing was carried out on an Illumina NovaSeq platform that generated 150 bp paired-end raw reads.

Raw reads generated by paired-end sequencing were cleaned by removing sequences with Illumina adapters, filtering out reads with N and reads that half of the bases with a quality score below 20. Then the high-quality mRNA reads were mapped back to the *Oncorhynchus mykiss* genome (ftp://ftp.ncbi.nlm.nih.gov/genomes/all/GCF/002/163/495/GCF_002163495.1_O-myk_1.0/) using Hisat2 (version 2.1.0) (31). Counts of each gene were extracted from the mapping files using Cufflinks (version 2.2.1) (32). Only the uniquely mapped genes which more than 10 reads in three or-more individual libraries were applied to differential expression genes' (DEGs) analyses using DESeq2 package (33). Finally, DEGs with adjusted *P* ≤ 0.05 and |log_2_ (fold-change)| ≥1 were considered as the targets for further analyses.

### Bacterial 16S rRNA Sequencing and Taxonomic Analyses

When sampling materials for microbiota analysis, skin tissue together with mucus were taken in account. Skin pieces with mucus were collected and mixed from trout head above the gill cover, back of the body below the dorsal fin, belly under the lateral line, and end of the body in front of the tail fin. About 200 mg skin sample was collected and homogenized by bead beating for 2 min at 60 Hz. Both fish and bacterial total genomic DNA were extracted following the manufacturer's recommendations of a DNA Min Kit (Qiagen, USA) and assessed photometrically using a NanoDrop ND-1000 spectrophotometer (Thermo Scientific). The universal primer set 515F (5′ -GTGCCAGCMGCCGCGGTAA-3′) and 806R (5′-GGACTACNNGGGTATCTAAT-3′) was used for the amplification of the V4 hypervariable region of bacterial 16S rRNA genes. After preparation and generation, the 16S rRNA libraries were sequenced on Illumina HiSeq platform 2500 at Novogene Bioinformatics Technology Co., Ltd, Beijing, China.

The open-source software system Quantitative Insights into Microbial Ecology (QIIME) quality filters were used to conduct raw data (34). Then these filtered sequences in the samples were picked and clustered into operational taxonomic units (OTUs) at an identity threshold of 97%. Ribosomal Database Project (RDP) classifier was used for the taxonomic assignment. The number of OTUs present in the samples was determined and calculated the richness of Chao1 and indexes of Shannon diversity in species-level. Student's *t*-test was used to estimate differences in alpha diversity (observed species and Shannon index). The skin microbiota of each groups were defined and analyzed by identified the OTUs using eulerAPE (http://www.eulerdiagrams.org/eulerAPE/). Beta diversity and taxon composition were analyzed by QIIME for calculating both weighted and unweighted UniFrac (35). The weighted UniFrac phylogenetic distance metric was analyzed by Principal Coordinate Analysis (PCoA) to further visualize variations of community members and structure. We performed multiple response permutation procedure (MRPP) which provides a test of whether there is a significant difference between control and infected groups of sampling units. To find the biomarkers statistical significant among different groups, we present the linear discriminant analysis (LDA) effect size (LEfSe) method to support high dimensional class comparisons with a particular focus on metagenomic analysis. All statistical significance in this study was set at a *P* < 0.05.

## Results

### Ich Infection Induced Morphological Changes and Immune Genes Expression in Trout Skin

**Figure 1:**
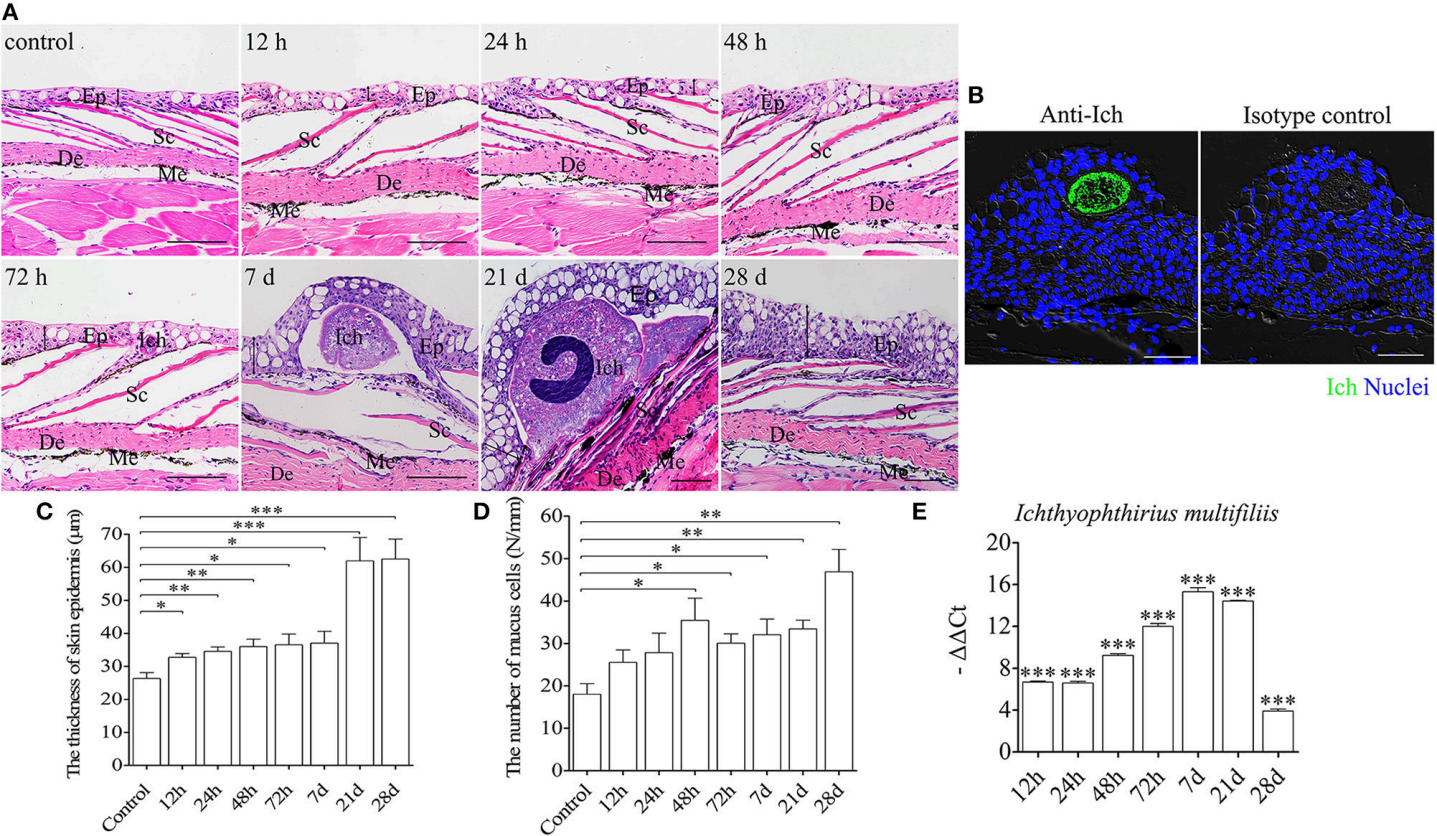
Pathological changes in skin of trout following Ich parasites infection. **(A)** Histological examination (hematoxylin/eosin stain) of skin from trout infected with Ich after 12, 24, 48, 72 h, 7, 21, 28 d and control fish (*n* = 6 fish per group). **(B)** Immunofluorescence staining for Ich (green) in skin paraffin sections from trout infected with Ich after 7 d is shown in the left, and the right panel display staining of trout skin with isotype control antibodies for anti-Ich. Differential interference contrast images (DIC) of skin are stained with the DNA-intercalating dye DAPI (blue) (*n* = 6 fish per group). **(C)** The thickness of skin epidermis of control and infected fish (*n* = 6 fish per group). **(D)** The numbers of mucus cells per millimeter in skin tissue of control and infected fish (*n* = 6 fish per group). **(E)** The expression of Ich-18SrRNA in trout skin was detected by qRT-PCR. The data was shown as –ΔΔCt. Skin structure is always displayed with the outside part of the epidermis (Ep) and the dermis (De). Scales (Sc), and melanophores (Me) are also indicated with black letters. The black line with double arrowheads indicates thickness of skin epidermis. Scale bars, 50 μm. ^*^P < 0.05, ^**^P < 0.01, ^***^P < 0.001 (unpaired Student's *t*-test). Data are representative of three different independent experiments (mean ± s.e.m.).

To evaluate morphological changes and expressions of immune-related genes in the trout skin, we exposed fish to a parasite, *I. multifiliis* (Supplementary Figure 1A), which elicits strong immune responses in mucosal tissues such as skin and gills (3, 36). By H & E staining and anti-Ich antibody detection, Ich parasites were easily detected in the skin epidermis of trout after infection (Figures 1A,B). Besides, morphological changes were also observed in the skin epidermis of trout. Histological studies exhibited significant increase of both the thickness of the skin epidermis and the number of mucus cells in the trout skin after infection (Figures 1C,D). Interestingly, the thickness of trout skin epidermis was found to be significantly increased at 12 h post infection, and remained relative stable up to 28 d (Figure 1C). In addition, we discovered that a significantly increased number of mucus cells in skin epidermis occurred at 48 h after Ich infection, with a continued increase up to 28 d (Figure 1D and Supplementary Figure 2). Moreover, many white dots were seen on the trout skin at 7 d after infection (Supplementary Figure 1B). Similarly, we detected the expression of Ich-18SrRNA in skin of trout after Ich infection and that of the control by qRT-PCR (Figure 1E). The result of high expression of Ich-18SrRNA in skin tissue further suggests that the parasite mainly succeeds in invading skin mucosal tissue.

**Figure 2:**
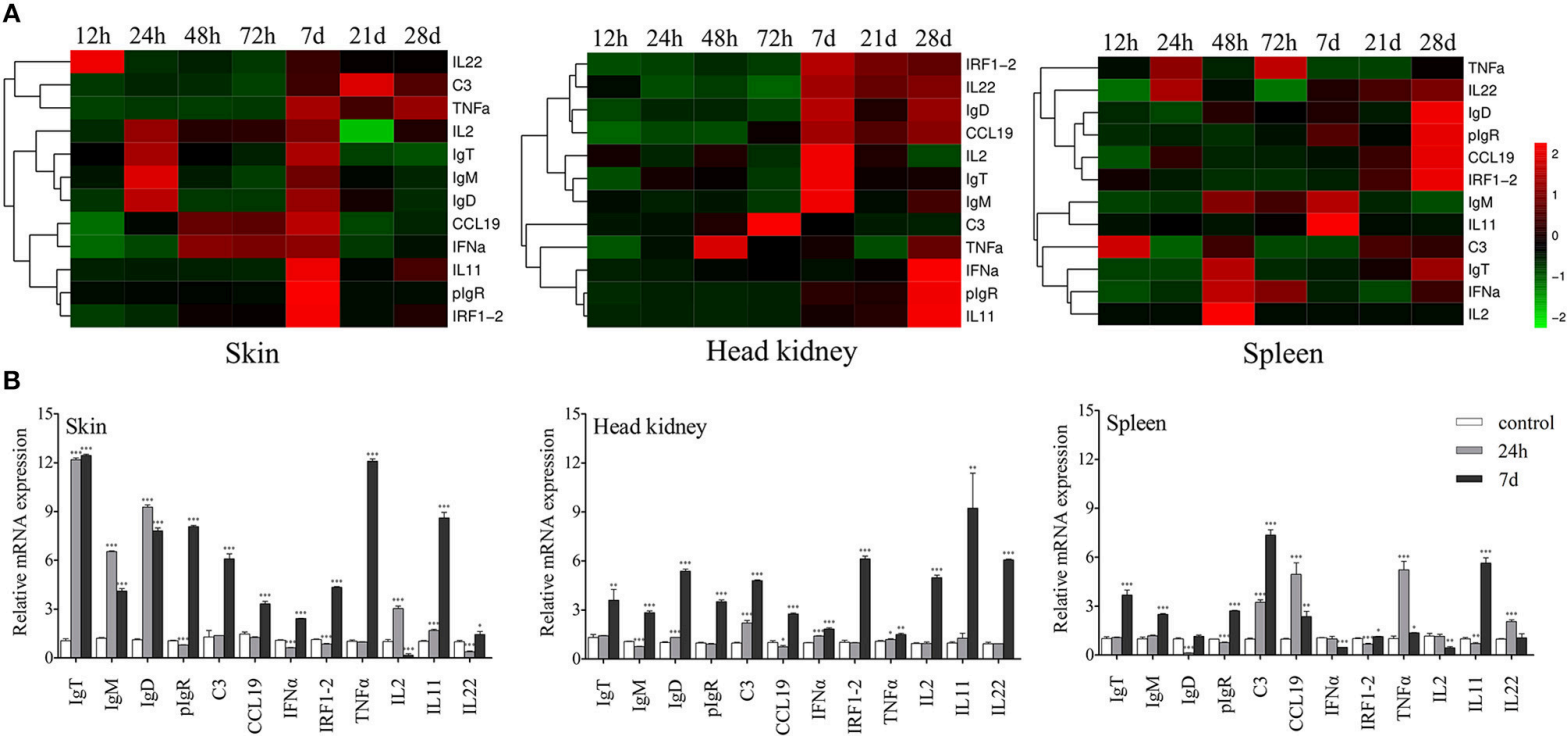
Kinetics of immune responses in skin of trout following Ich parasites infection. **(A)** Pheatmap package of R (version 3.4.4) was used to picture heat maps to illustrate the kinetics of immune responses in trout skin (left), head kidney (middle), and spleen (right) of Ich-challenged vs. control group measured at 12, 24, 48, 72 h, 7, 21, and 28 d after infections with Ich parasites (*n* = 6 fish per group). Pearson correlation was carried out and ‘Complete’ method was used to cluster values. **(B)** Fold changes of immune genes and makers in different tissues at 24 h and 7 d were shown with histogram. ^*^P < 0.05, ^**^P < 0.01, ^***^P < 0.001 (unpaired Student's *t*-test). Data are representative of three different independent experiments (mean ± s.e.m.).

To study the mRNA expression levels of immune-related genes and cell markers in trout skin after infection, we measured 12 immune-related genes including the cytokines (interleukin 2, 11 and 22; tumor necrosis factor α, interferon α, and interferon regulatory factor 1-2), chemokine gene (chemokine-like 19), complement 3 (C3), poly immunoglobulins receptor (pIgR), and immunoglobulin heavy chain genes (IgT, IgM, and IgD) (Figure 2A; primers used in this study are shown in Table 1) by qRT-PCR. Importantly, our studies discovered that strong immune responses occurred in the trout skin, head kidney, and spleen after challenge with Ich parasites. After infection, significantly up-regulated mRNA expressions of immune-related genes were detected in trout skin as early as 12 h (for IL22) or 24 h (IL2, IgT, IgM, and IgD) and reach their peak levels at 7 d, then recovered to control levels by 28 d (Figure 2B). While in the trout head kidney and spleen, delayed immune responses were discovered after challenge with Ich parasites, except TNFα at 48 h in head kidney and C3 at 12 h in spleen (Figure 2B). Interestingly, days 1 and 7 were the most relevant in terms of the intensity of the immune response in skin; therefore, these two time points were selected for subsequent RNA-Seq analysis.

### Analysis of Transcriptomic Changes in Trout Skin After Ich Infection

For further analysis of the two time points mentioned above, RNA-Seq libraries made from nine samples which separately represent three groups (control, E24h, and E7d) were sequenced on the Illumina platform. A total of 247,233,899 paired-end raw reads were generated. After filtration, 241,428,084 high-quality reads were harvested (Supplementary Table 1). The total clean reads were mapped back to *O. mykiss* sequences, and the mapped rate of each sample was over 80 percent (Supplementary Table 2). Unique mapped genes were further filtered, and only more than ten reads in three or-more individual libraries were used for differential expression genes (DEGs) analyses. As a result, the E24h/Con and E7d/Con groups yielded 27,536 and 27,692 valuable genes, respectively.

**Figure 3:**
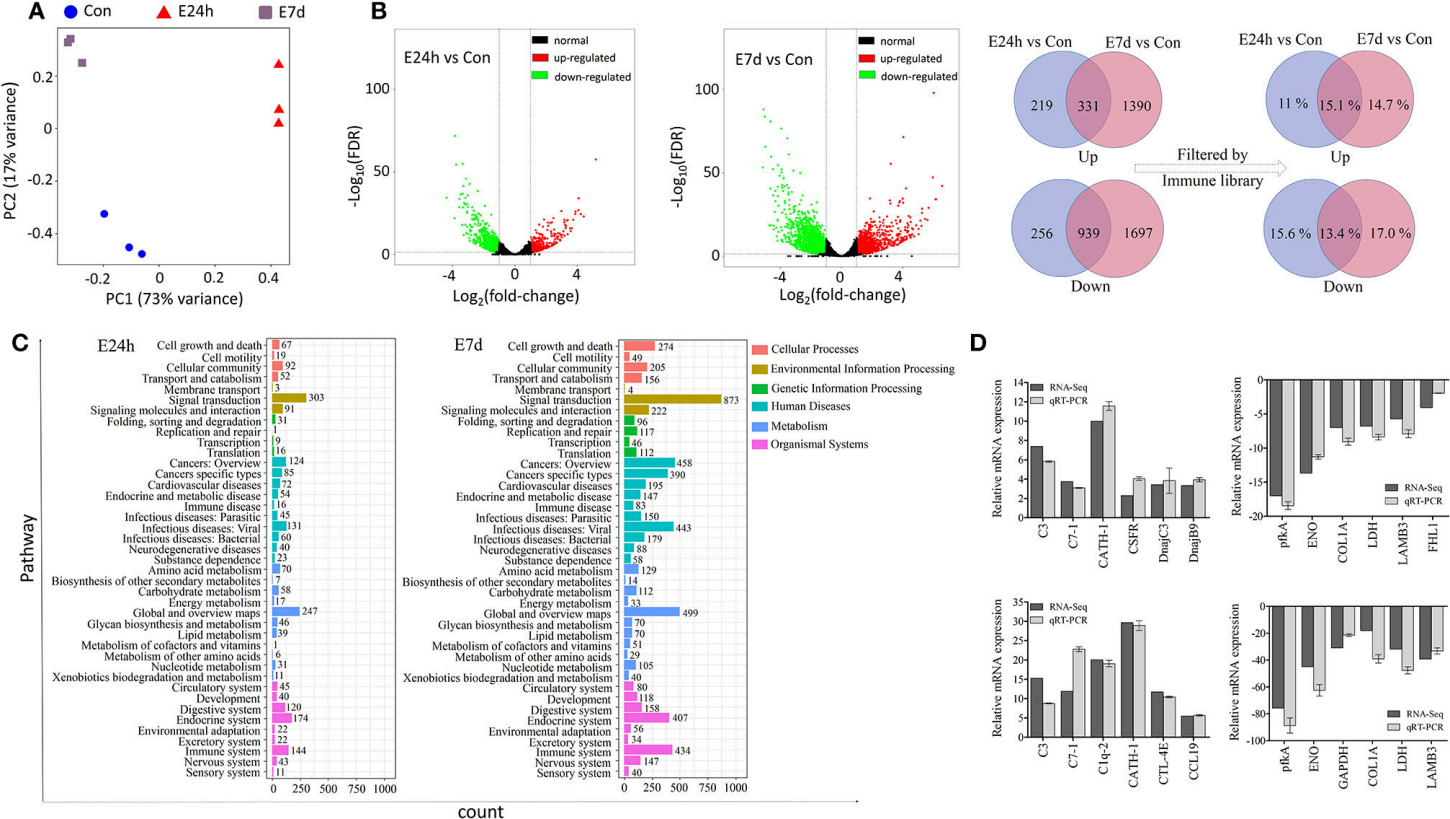
Effects of Ich-stimulation on the longitudinal transcriptomic dynamic changes of trout skin. **(A)** Principal component analysis (PCA) of the skin transcriptome in basal conditions and Ich-infected groups. Plum squares represent control samples, red triangles are treated samples at 24 h and blue circles are experimental samples at 7 d after Ich infection. **(B)** Volcano plot displaying the DEGs distribution in each experimental group compared with control group. Venn diagram showing the number of up- and down-regulated differential genes and the percentage of immuno-related genes among them after filtered by immune library. Con, control skin; E24h, 24 h post-infected skin; E7d, 7 d post-infected skin; Red spots, expression fold change of >2 and FDR of <0.05; Green bots, expression fold change of <2 and FDR of <0.05; Black spots, no difference in expression. Up, up-regulated differential genes; Down, down-regulated differential genes; Light blue, genes that were significantly regulated at 24 h; Rose, genes that were significantly regulated at 7 d; Lilac, genes that were significantly regulated at both time points. **(C)** The number of DEGs in each KEGG pathway. Different colors represent different categories. **(D)** Transcriptomic differential expressed genes in experimental groups were detected using qRT-PCR to validate RNA-seq. Positive numbers in the Y axis mean up-regulated, while negative values mean down-regulated.

Prior to DEGs analysis, the filtered genes of each individual library were used to generate a PCA plot for principal component analysis (Figure 3A). As shown in the plot, there was a significant difference among the three treatment groups. During the statistical analysis, 1,745 DEGs were identified in the E24h/Con group (550 up-regulated and 1,195 down-regulated) and 4,357 DEGs in the E7d/Con group (1,721 up-regulated and 2,636 down-regulated). The distribution trends of DEGs in the pairwise comparisons (E24h/Con, E7d/Con) were presented as volcano plots (Figure 3B). From these plots, it is striking that there were much more up-regulated genes in the E7d than in the E24h group when compared with controls. After filtering by the *O. mykiss* immune gene library, more than 10 percent of DEGs indicated immune genes as shown in the Venn diagram (Figure 3B, right). What is noteworthy is that 14.7% immune genes were only significantly up-regulated in the E7d/Con group, while 15.1% immune genes were significantly up-regulated at both time points. To validate the RNA-Seq expression levels, we randomly detected six up-regulated and six down-regulated immune genes' expression levels by qRT-PCR. The relative mRNA expression values determined by RNA-Seq and qRT-PCR were significantly correlated (Figure 3D), suggesting that RNA-Seq is as sensitive and accurate as qRT-PCR for determining gene expression *in vivo*.

**Figure 4:**
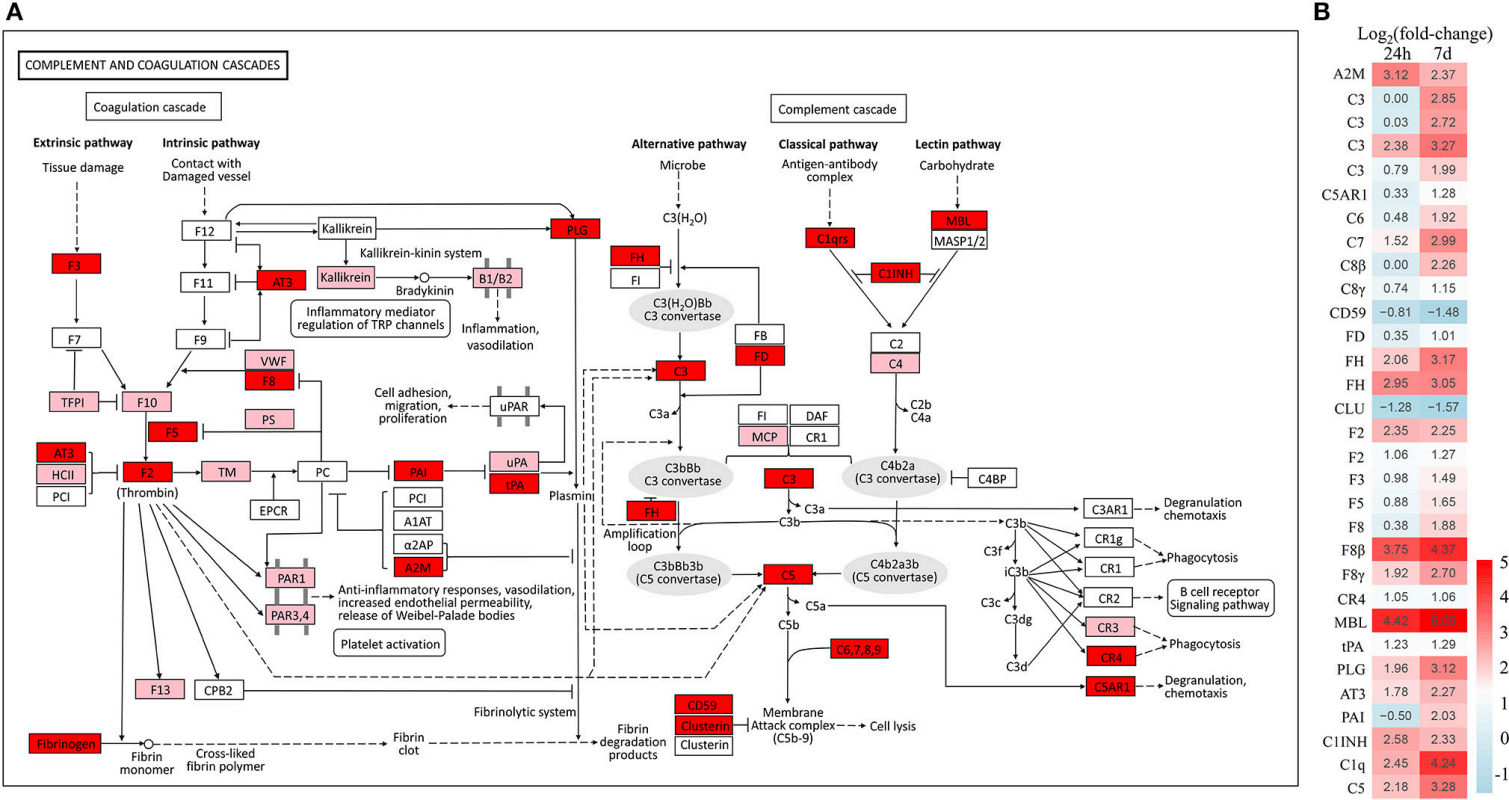
Overview of the complement and coagulation cascades pathway in trout skin. **(A)** Both of red and pink shading boxes represent molecules of the complement and coagulation cascades pathway identified in trout skin. And the red boxes indicate DEGs in this pathway. The figure is modified from KEGG map04610. **(B)** Differential expression genes involved in the complement and coagulation cascades pathway were analyzed at 24 h and 7 d post exposure to Ich. The color gradient represents highly up-regulated (red) to highly down-regulated (green) genes.

Based on the common immune genes of the two aforementioned time points, we selected 20 genes with high differential expression. As shown in Table 2, most of the up-regulated immune genes belonged to innate immune molecules including lectin, mucins, pro-inflammatory cytokine, antimicrobial peptides, complement activation cytokines, and so on. In addition, complement and coagulation cascades as well as toll-like receptor (TLR) signaling pathways with their heat maps were generated to demonstrate the differential genes' expression patterns (Figure 4 and Supplementary Figure 3, respectively). Interestingly, there is no significant change of TLR expression. However, pro-inflammatory and chemotactic molecules (IL-1, IL-8) were up-regulated dramatically at both 24 h and 7 d. Moreover, some cytokines in connection with T cells (CD40, MIG) were also up-regulated markedly in the E7d/Con group. This indicates that adaptive immune response might be activated after 7 d post infection. Furthermore, almost all of the significantly different genes in complement and coagulation cascades pathways were up-regulated, suggesting that the complement components play a crucial role in anti-parasitic invasion.

**Table 2:** The 20 up- and down- regulated immune genes.

**Gene name**	**GenBank** **accession no**.	**Description**	**E24h/Con**		**E7d/Con**	
			**Log2FC**	**FDR**	**Log2FC**	**FDR**
CATH-1	NM_001124480.1	Cathelicidin-1	2.60740	8.18E-13	4.216072	1.81E-41
Lectin	NM_001124514.1	Lectin	3.25396	1.15E-16	6.180577	6.91E-35
HP	XM_021595153.1	Hepcidin-like	−1.29474	0.003033	3.820614	7.83E-34
APOA2	NM_001161448.1	Apolipoprotein A-II	3.857831	1.13E-15	4.087136	1.17E-34
MMP13	XM_021618131.1	Collagenase 3	0.988225	0.012413	4.02495	7.83E-33
MRC	XM_021588692.1	Macrophage mannose receptor 1-like	2.51501	1.04E-15	3.194697	2.43E-32
HSP90α	XM_021600916.1	Heat shock protein HSP 90-alpha	1.682875	9.04E-09	3.337275	3.88E-21
IL8	XM_021625343.1	permeability factor 2	1.017484	0.021847	2.845917	2.78E-16
HP	XM_021586023.1	Haptoglobin-like	3.282396	6.24E-18	4.183853	3.51E-16
MUC-5AC	XM_021607524.1	Mucin-5AC-like	−1.36918	5.15E-05	−2.72499	3.32E-15
CTL	XM_021562202.1	C-type lectin	0.874360	0.009486	2.939317	1.78E-15
APOA1	NM_001124247.1	Apolipoprotein A-I	4.075171	1.65E-27	3.613799	3.2E-13
CFH	NM_001124410.1	Complement factor H	2.064596	1.17E-07	3.167978	1.45E-13
C1q	XM_021620193.1	Complement C1q-like	2.453538	2.82E-06	4.240708	9.51E-11
HSP90β	NM_001124745.1	Heat shock protein 70 beta	2.760112	1.38E-14	2.785039	1.5E-11
CLEC4E	NM_001160495.1	C type lectin receptor beta	1.333084	0.000051	3.689057	2.57E-24
MUC-2	XM_021562134.1	Mucin-2-like	1.40047	0.001738	−3.08191	5.23E-08
MUC-5B	XM_021580807.1	Mucin-5B-like	2.047236	0.000002	1.642672	0.00079
RA	XM_021604719.1	Retinoic acid receptor responder	−0.69702	0.200366	−1.4504	0.001658
MUC-17	XM_021610915.1	Mucin-17-like	1.294894	0.008125	1.424968	0.010883

To further investigate the DEGs which were involved in responding to Ich infection among the three groups, KEGG pathway enrichment analysis was conducted (37). As is shown in Figure 3C, both the signal transduction category and immune system category which were most related to immune response had an enormous advantage in gene count. In addition, 1,031 DEGs had KEGG Ortholog annotation; 618 DEGs had pathway annotation in the E24h/Con group, but only 7 pathways were significantly enriched (*P* < 0.001), including complement and coagulation cascades pathways (Supplementary Table 3). In the E7d/Con group, 2,677 DEGs had KEGG Ortholog annotation, 1,582 DEGs had pathway annotation, and 33 pathways were significantly enriched (*P* < 0.001) containing pathways associated with immune response, signal transduction, infectious disease, and cancer. Moreover, some protozoan parasite disease pathways were significantly enriched in the E7d/Con group, including amoebiasis and malaria (Supplementary Table 3). These results further suggest that the DEGs of the complement system play a crucial part in defense against parasite invasion and that a stronger immune response to Ich had occurred in the trout skin of E7d/Con group.

### 16S rRNA Sequencing and Taxonomic Analyses

We investigated the abundance and diversity changes of trout skin microbiota communities in response to Ich infection using 16S rRNA sequencing of skin samples from control and infected fish by Illumina MiSeq run. We obtained 1,369,039 raw reads from the original samples collected at time intervals of 24 h and 7 d after Ich infection. Importantly, we developed a new protocol, facilitated by the Quantitative Insights Into Microbial Ecology (QIIME) toolkit (34), that can perform standard microbial community analysis techniques, including the quality filtering of reads, efficient operational taxonomic unit (OTU) picking, taxonomy assignment, the computation of α and β diversity measures, and other analyses.

After the criteria filtering of raw reads, 1,293,982 high-quality clean reads were acquired in total and these were used for downstream analyses. The mean number of post-filter reads per sample was 76,057 that were exactly 252 nucleotides in length respectively. The sequences were divided into unique OTUs at the 97% level using CD-HIT (38), which is the standard tool for this task when handling pyrosequencing data. Then 21,032 OTUs were noted and the total number of OTUs detected in experimental groups (E24h: 9,282 OTUs; E7d: 6,977 OTUs) were much higher than in the control groups (4,773 OTUs) of skin samples (Supplementary Figure 4). The data in our results indicated that the trout skin microbial community may have been altered by Ich infection.

### Ich Infection Results in Skin Microbial Dysbiosis

**Figure 5:**
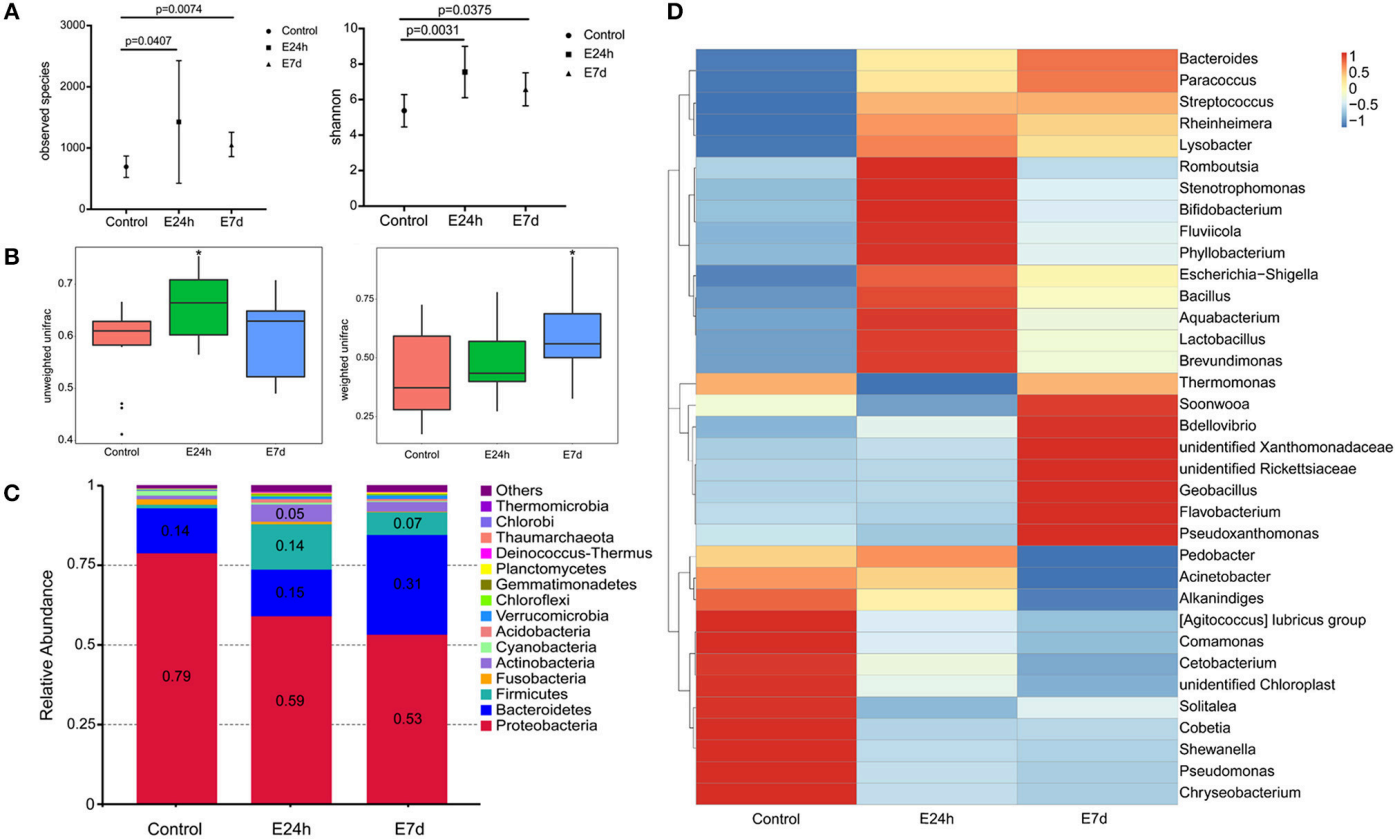
Changes in the abundance and diversity of trout skin microbiota community in response to Ich parasites infection. **(A)** Microbial alpha diversity was destabilized post Ich infection. Calculating of observed species and Shannon index from different groups indicated significantly increased alpha diversity at 24 h and 7 d post infection. **(B)** The microbial beta diversity was analyzed using unweighted Unifrac and weighted Unifrac also exhibited destabilization after Ich infection. **(C)** Bacterial community composition of trout skin in each group was extremely changed after parasite stimulation. Bar chart of the mean relative abundance in phyla was presented to describe the details. Numbers that had abundances >0.05 (5%) were just noted to represent the relative percentage of each phyla. **(D)** Heat map showing the relative abundance reveals striking microbial dynamics and compositional differences between control and infected groups based on top 35 OTUs in the skin. Columns are arranged by similarity using hierarchical clustering. The relative abundance data was log 10 transformation. E24h, 24 h after Ich infection; E7d, 7 days after Ich infection. ^*^P < 0.05 (unpaired Student's *t*-test) in figure B.

We calculated the differences in the microbial diversity and community in trout skin between the experimental and control groups by using the OTUs noted above for further analysis with UniFrac. Interestingly, high alpha diversity values of microbiota in trout skin from the experimental groups were discovered at both 24 h (Student's *t*-test, *P* = 0.0407 for observed species and *P* = 0.0031 for Shannon index) and 7 d (*P* = 0.0074 for observed species and *P* = 0.0375 for Shannon index) after Ich infection when compared with the control group (Figure 5A, Table 3). In addition, these changes were examined in beta diversity analysis using unweighted UniFrac and weighted UniFrac, and significant differences were observed at 24 h (Figure 5B, left for the unweighted UniFrac value) and 7 d (Figure 5B, right for the weighted UniFrac value). Thus, our result indicated that Ich infection could induce an increase of microbial diversity in trout skin.

**Table 3:** Alpha diversity metrics (mean ± standard deviation) of the rainbow trout skin microbial community.

**Group**	**Observed species**	**Shannon**	**Simpson**	**Chao 1**	**Ace**	**Goods coverge**	**PD whole tree**
Con	694	5.372	0.92	870.29	883.019	0.995	69.198
E24h	1,426	7.553	0.972	1579.935	1578.582	0.994	123.371
E7d	1,050	6.582	0.932	1179.393	1175.885	0.996	100.089

We identified bacteria changes in Ich-infected individuals by classifying the phylum, class, order, family, and genus of the microbial sequences in trout skin from control and infected groups. The most abundant phyla across all samples were *Proteobacteria* followed by *Bacteroidetes, Firmicutes*, and *Actinobacteria* (Figure 5C). At the phylum level, the mean abundance of *Proteobacteria* dropped from 79% in control to 56 ± 3% in infected groups. Meanwhile, the abundance of *Firmicutes* present increased from 1% in control fish to 14 and 7% in the 24 h and 7 d experimental groups, respectively. More importantly, an increased abundance (2.2-fold) of *Bacteroidetes* in trout skin was detected at 7 d after Ich infection. Additionally, the abundance of *Actinobacteria* was observed to increase (4.9-fold) as a result of Ich infection at 24 h (Figure 5C).

Details about the changes in the microbial community at the family and genus level were shown in a heat map for the top 35 abundant OTUs with significantly different abundances in each infected group (Figure 5D). Similar to the microbial abundance detected at the phyla level above, 12 OTUs, including the *Moraxellaceae, Shewanellaceae*, and *Pseudomonadaceae* family that belong to the phylum *Proteobacteria*, were detected with much higher abundances in the control group than the infected groups. While in the E24h group, the phylum *Firmicutes* and *Actinobacteria* showed higher relative abundance than that in controls. Importantly, at seven days after Ich infection, the relative abundance of the family *Flavobacteriaceae* that belongs to *Bacteroidetes, Bdellovibrionaceae*, and *Rickettsiaceae* that belong to *Proteobacteria* were much higher than the other two groups.

### Biomarkers of the Bacterial Community in Different Groups

**Figure 6:**
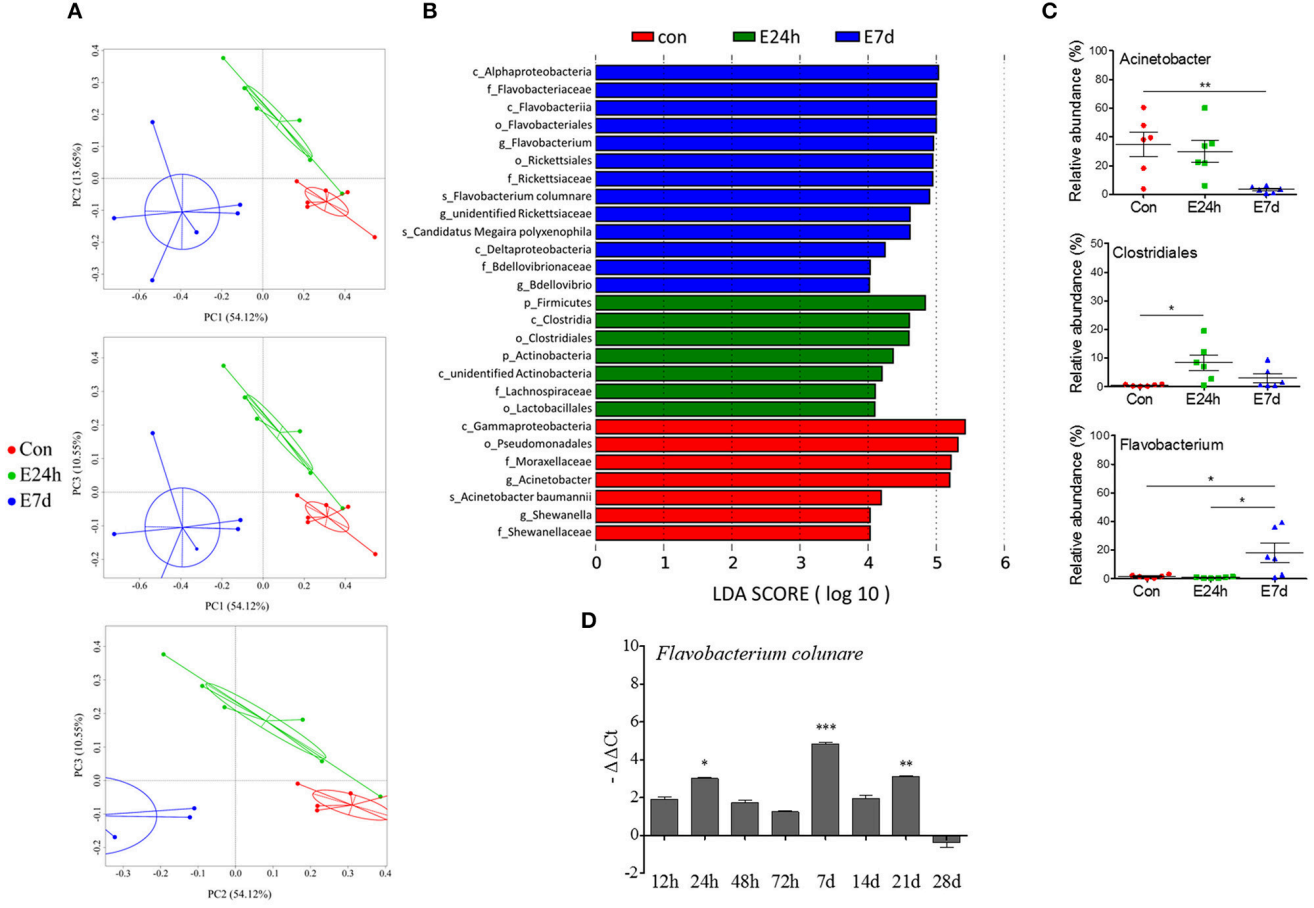
The longitudinal variation of microbial composition and biomarkers following Ich-parasites invasion. **(A)** Principal coordinate analysis (PCoA) with weighted UniFrac distance matrix for all the 18 taxonomic profiles from trout skin following parasite infection. Each dot represents one sample. Red pots indicate control samples, green for E24h and blue for E7d, respectively. **(B)** Description of biomarkers that were significant different between control and infection groups. **(C)** Ich infection results in losses of beneficial bacteria and increased abundance of opportunistic pathogens in trout skin. Percentage of total OUTs represented by *Acinetobacter, Clostridiales*, and *Flavobacterium* in control and infected groups were shown. **(D)** Relative abundance of *Flavobacterium columnare* shown as –ΔΔCt was increased according with Ich infection in experimental groups. Con, control group; E24h, 24 h after Ich infection; E7d, 7 days after Ich infection. ^*^P < 0.05, ^**^P < 0.01, ^***^P < 0.001 (unpaired Student's *t*-test). The qRT-PCR data in **(D)** were representative of three different independent experiments (mean ± s.e.m.).

Despite the findings that differences both of skin microbial communities' diversity and abundance were existed between the control group and experimental group, PCoA showed that Ich-infected samples clustered apart from each other and that the distances between control and infected groups were greater at 7 d than those at 24 h (Figure 6A). In addition, we determined whether Ich infection resulted in loss of important normal flora bacteria or increased opportunistic bacteria.

LDA Effect Size (LEfSe) analysis was used to further study the microbial biomarkers that contribute to the bacteria changes in different groups. Among the biomarkers, we observed that the abundances of *Acinetobacter* that belong to gammaproteobacteria phyla were significantly decreased at 7 d after infection (Figures 6B,C). Additionally, another two members of gammaproteobacteria phyla, which were the *Shewanella* and *Pseudomonas* genera were practically absent in infected samples (Supplementary Figure 5). In contrast, *Clostridiales, Actinobacteria*, and *Lachnospiraceae* scarcely existed in the control groups but were increased in the infected groups. We also found significantly increased abundances of *Flavobacterium, Rickettsiaceae*, and *Bdellovibrio* at 7 d after infection (Figure 6C, Supplementary Figure 5). Among the *Flavobacterium* members, *F. columnare* (*F. columnare*), which is well known as a bacterial pathogen that directly invades fish skin and gills and causes heavy infection, was significantly increased in the skin microbial community of group E7d. Meanwhile, the relative abundance of *F. columnare* in trout skin was examined using qRT-PCR, and the increased presence of this pathogen was observed along with Ich infection (Figure 6D). However, it is difficult to detect both *F. columnare* (Figure 6D) and Ich (Figure 1E) in the trout skin from the control group. Taken together, our results first suggest that Ich parasite infection results in trout skin microbial dysbiosis.

## Discussion

The skin is one of important mucosal surfaces and immune barriers in teleost fish, which represent the most ancient bony vertebrates that contain a SALT (3, 30). Due to the aquatic environment, the teleost skin surface coexists with millions of diverse microorganisms. Moreover, these microbiota and their products both directly and indirectly shape the skin mucosal system of teleost fish (39, 40). Although the interactions between commensal microbiota and the teleost mucosal immune system are known to be critical, however, the links between the teleost skin immune process and microbial dysbiosis after parasitic infection are poorly understood. To our knowledge, this study is the first time to show the transcriptome and microbiome in the skin of rainbow trout after challenged with the Ich parasites.

Here, we evaluate the pathological and morphological changes in trout skin after exposure to Ich. Although the entry route of Ich is currently unclear, we found the Ich parasite existed in the skin epidermis by H & E staining, which indicated that Ich has successfully invaded the trout skin mucosal tissue. With the occurrence of infection, significantly increased number of mucus cells and thickness of epidermis were observed in trout skin. In agreement with the significant histopathological lesion in trout skin, we found that 12 immune-related genes were significantly activated in trout skin as early as 12 or 24 h when compared with those in head kidney and spleen after Ich infection. Our data probably suggest that teleost skin, as the first line to defense against Ich infection, activates the immune responses much earlier than that in systemic immune tissues such as head kidney and spleen. Importantly, at 7 d after infection, both the innate and adaptive immune molecules were responsive to Ich in trout skin. Future studies will investigate the specific contributions of adaptive immune molecules such as IgM, IgD, and IgT at the early stage to the skin immune response in teleost fish.

We used the next-generation sequencing based whole transcriptome analysis tool RNA-seq to characterize the details of all gene expression levels comprehensively at 24 h and 7 d in trout skin after Ich infection. The RNA-seq based expression studies provided a general view of the biological processes that took place in the trout skin as a result of Ich infection. In total, 241,428,084 high-quality reads were harvested and mapped to unique locations in the *O. mykiss* sequences. By statistical analysis, we identified 1,745 DEGs in the E24h/Con group and 4,357 DEGs in the E7d/Con group. These data further suggest that the immune response of trout skin to Ich infection was stronger and more extensive in the E7d/Con than E24h/Con group according to the results we obtained by qRT-PCR. Importantly, our study has identified SALT-related immune molecules that respond to Ich, including cytokines, complement factors, and antibacterial peptides, all of which are known to be crucial in teleost's skin immune system. Below, we highlighted several key constituents of these categories and their potential functions in the context of trout skin responses to Ich.

Our RNA-Seq results showed increased expression levels of almost all significant DEGs in the complement pathway. As a bridge that connects innate and adaptive immunity in vertebrates, the complement system plays a pivotal role in the defense against pathogen invasion (41, 42). Previous study reported that the complement system of fish can be induced by either bacterial or parasitic infection (43, 44). In this study, 10 complement-related functional genes in total, including C1q, C3, C5, C6, C7, C8β, C8γ, MBL, FH, and FD, were activated following Ich infection in trout skin. Meanwhile, the membrane-bound complement regulatory protein (CD59) that acts to limit the assembly of membrane attack complex (MAC) and protects the body's tissue cells from accidental damage by the complement system (45, 46), was significantly down-regulated in trout skin after challenge with Ich parasites. These results indicate that an intense response of complement factors to pathogens occurred in trout skin. Thus, the successful invasion of Ich both damaged the skin mucosal tissue and caused drastic reactions relating with complement in rainbow trout.

Moreover, our study found that the expression levels of pro-inflammatory cytokines (IL-1β, IL-8, and TNF-α) were up-regulated after Ich infection. A previous study proved that cytokines are always produced at the pathogen entry site; they can drive inflammatory signals to regulate the capacity of residents and newly arrived phagocytes to destroy invading pathogens (47). As an important member of the pro-inflammatory cytokines, IL8 is barely noticeable in healthy tissues but can be rapidly induced by different inducers such as IL-1β, TNF-α, bacteria, viral products, and cellular stress (48, 49). Tumor necrosis factors (TNFs) are inflammatory cytokines that induce several immunological responses, including the apoptosis of infected cells (50). Combining previous studies, our results suggest that parasitic infection has induced drastic inflammatory reactions in trout skin.

In addition, other cytokines connected with T cells (CD40 and MIG) were also found to have markedly up-regulated expressions in the E7d/Con group while there was no significant change in TLR expression. Cytokines can also regulate antigen presentation function in dendritic cells, and their migration to lymph nodes to initiate the adaptive immune response in mammals (47). Thus, we further speculate that Ich invasion may activate the adaptive immune system in trout skin.

Interestingly, significantly increased expression levels of antimicrobial peptides (hepcidin and cathelicidin-1) were detected in trout skin after parasite infection through RNA-Seq analysis. A previous study showed that hepcidin can protect grass carp against *F. columnare* infection via regulating the iron distribution and immune gene expressions (51). In teleost, cathelicidin can modulate the function of macrophages via the P2X7 receptor (52). We suppose that antimicrobial peptides (hepcidin and cathelicidin-1) may also play important roles in responding to parasite infection in trout skin. In addition, we hypothesize that the up-regulated genes here, which were previously proved to be mainly involved in antimicrobial immunity in teleost skin, are activated to combine with the increased bacterium after Ich infection.

In this study, we performed 16S rRNA sequencing that revealed important shifts in the skin bacterial community composition of rainbow trout in response to Ich infection. As mucus layer coated on fish skin is part of the skin associated mucosal system, fluctuations of the microbiota community load on trout skin and skin mucus were detected after Ich infection. We observed the differences of diversity indexes between the baseline skin microbiomes of Ich infected subjects and normal healthy individuals. In general, our data corroborate previous studies in terms of diversity changes after the pathogen infection (4, 16, 53). However, we found that the experimental groups had statistically significant higher values than the control group for the alpha diversity index, which was not absolutely identical to previous research (10). Interestingly, a recent study of Atlantic salmon showed no significant changes in the skin microbiome diversity (alpha diversity) of control and virus-infected fish due to high inter-individual variability (4). Moreover, increased diversity in the skin microbiome was observed in the evaluation of microbiota in both wild tropical fish skin and their parasitic loads (54). Thus, considering previous studies, our results indicate that the impact of pathogens on the fish microbiota diversity may vary between species and pathogens.

Previous study of Atlantic salmon indicated that fluctuations in environmental microbiota did not seem to be the root cause of skin microbiota differences after infected with salmon lice. Sampling point (time) as well as infection status are the drivers of microbiome community dynamics (53). Regarding community shifts, statistical analyses in our study similarly revealed that treatments and times after infection were important factors that could determine the composition of the skin microbial community in rainbow trout. *Proteobacteria* is the predominant phylum in the skin microbiome of teleost (10, 55, 56). The decrease of *Proteobacteria* abundance at 7 d after Ich infection in this study was primarily the result of the complete loss of *Acinetobacter*, a genus known to dominate the skin microbial community of trout. In addition, we discovered the absence of *Shewanella* and *Pseudomonas* of the *Proteobacteria* phylum in all Ich-infected groups regardless of infection time, which indicates that these commensal bacterial species were unable to recolonize the host for the duration of the parasite infection. Importantly, several skin *Proteobacteria* isolates from salmonids have inhibitory effects against bacterial and fungal pathogen infections (10, 55). Moreover, *Proteobacteria* also dominate the human skin microbiome (57) and play a major role in the management of opportunistic bacteria regulating the host–environment relationship (58). Therefore, although the specific physiological contribution of *Proteobacteria* in the trout skin is currently unclear, further studies should investigate whether the decreased abundances of *Acinetobacter, Shewanella*, and *Pseudomonas* in *Proteobacteria* can contribute toward differences in the disease susceptibility of trout upon Ich infection.

In contrast to the decreased abundance of *Acinetobacter, Shewanella*, and *Pseudomonas*, we observed the increased incidence and intensity of *Flavobacterium* in trout skin after parasitic infection for the first time, particularly in the E7d experimental group of the present study. Our results parallel the previous study that *Aeromonas salmonicida* infection was associated with dominance shifting to opportunistic pathogens on the skin of Atlantic salmon (20). Similarly, the absence of the majority of the *Proteobacteria* and increased abundances of opportunistic bacterial pathogen (*Vibrionaceae, Flavobacteriaceae*, and *Streptococcaceae sp*.) were also detected in the skin of virus-infected salmon, which indicated that pathogen infection could facilitate colonization by opportunistic bacteria (4). These results agree with the observed expansions of *Flavobacteriaceae* and other known opportunistic taxa mentioned above in Ich-infected trout skin following lowered *Proteobacteria* abundance in our research. Thus, our results suggest that the increased abundance of *Flavobacteriaceae* could be opportunistic bacterium that may cause secondary infections in trout skin after parasite invasion. Besides, pathogens such as *F. columnare* were also proliferated due to Ich infection. Importantly, since our observations show a high presence of *F. columnare* in Ich-infected fish, there must be a link between Ich infection and *F. columnare* invasion in trout skin. As is shown in our study, Ich infection could cause mechanical damage in trout skin. We make a hypothesis that the skin damage augment the adhesion of secondary pathogenic bacteria to epithelial surfaces. Thus, Ich infection possibly provide opportunities for *F. columnare* adhesion.

**Figure 7:**
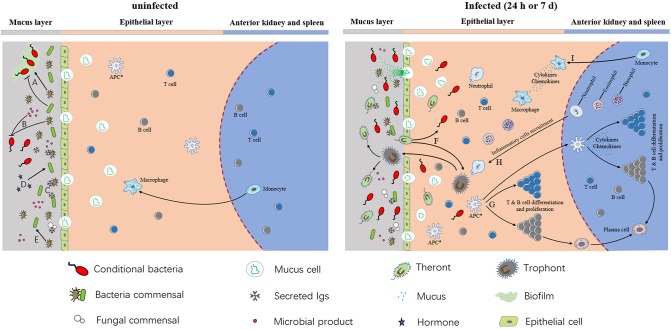
A model of interactions among host, microbiota, and pathogens in trout skin. (A) Commensal microbes can promote or inhibit biofilm formation. (B) Commensal bacteria and their products can inhibit conditional bacteria. (C) Secreted Igs coat on the surface of skin microbiota. (D) Host hormones have an effect on conditional bacteria. (E) Commensal microbes and their products can affect other commensal microbes. (F) The parasite invaded the organism and resulted in the colonization of opportunistic pathogens in teleost skin. (G) T & B cells differentiation and proliferation after APC presented the information of pathogen. The APCs in epithelial layer are hypothetical (indicated by asterisks). (H,I) Macrophages and inflammatory cells like neutrophils, basophils, eosinophils, and neutrophils were recruited to infected areas, and the pro-inflammatory molecules were generated in both infected epithelia and the central lymphoid tissues of the anterior kidney and spleen.

In conclusion, our results summarize as a model that parasite infection, pathological changes, host mucosal immune responses, and bacterial community composition occur in the skin of rainbow trout. As shown in Figure 7, commensal microbes coexist in normal trout skin and play a key role in maintaining the homeostasis and suppression of the conditional bacteria. The continuously produced mucus layer covers trout skin and contain molecules with immunologically important properties, which interact directly with commensal microbial populations at skin surface. Besides, the interactions among different skin commensal bacteria are also benefit for maintaining the homeostasis. However, our study indicates that Ich infection destroyed both the homeostasis and the mucous layer. The successful invasion of Ich results in significant histopathological lesions in trout skin, which in turn provide much more chances for Ich and other pathogens' infection. To deal with the disadvantage environment, mucus cells increased and released much more mucus to enhance physical or chemical barrier, and skin mucosal immune response are also induced at the same time. Our data first demonstrated that Ich infection results in dramatic immune responses, including inflammatory reactions, complement reaction, and bactericidal effects, and microbial dysbiosis, characterized most prominently by the decrease of *Proteobacteria* in trout skin. As for innate and adaptive immune response, studies of channel catfish and rainbow trout have shown that T and B cells are presented in fish skin and can respond to the protozoan parasite *I. multifiliis* infection (3, 59, 60). Neutrophils could surround the parasite *I. multifiliis* after 2–3 days post-infection and by 5–6 days inflammatory cells including neutrophils, eosinophils, and basophils can be recruited to infected areas of juvenile carp skin (61). In our study, pro-inflammatory cytokines such as IL8, IL-1α, and TNF-β are significantly up-regulated and these molecules play a role in the recruitment and maintenance of inflammatory cells in the rainbow trout skin after *I. multifiliis* infection (62). Furthermore, our results indicate that the loss of microbial balance caused by parasite infection likely impacts the health of the fish host, rendering the fish more susceptible to subsequent infections caused by the opportunistic pathogens and other bacterial pathogens present in the environment. These findings provide a critical basis for understanding the complexity of the interactions among pathogens, microbiota, and hosts in teleost fish. Future studies should address how the defensive mechanisms of specific immune responses in trout skin against Ich infection correlate with the observed changes in the skin microbiome of teleost fish.

## Ethics Statement

All animal procedures were approved by the Animal Experiment Committee of Huazhong Agricultural University (HZAUFI-2016-007) and carried out according to the relative guidelines.

## Author Contributions

XiZ and LD performed most of the experiments, analyzed the data, and wrote the manuscript. YoY, WK, YaY, and ZH help with most of the experiments. XuZ contributed to setting up the Ich infection model. ZX designed the experiments and revised the manuscript. All authors reviewed the manuscript.

### Conflict of Interest Statement

The authors declare that the research was conducted in the absence of any commercial or financial relationships that could be construed as a potential conflict of interest.
