# Supplementary material for: The Change of Teleost Skin Commensal Microbiota Is Associated With Skin Mucosal Transcriptomic Responses During Parasitic Infection by Ichthyophthirius multifillis
Source: Front Immunol. 2018 Dec 18;9:2972. doi: 10.3389/fimmu.2018.02972 (PMC6305302; doi:10.3389/fimmu.2018.02972)
Supplement: Supplementary file 1 [file Presentation_1.pdf]

Title:

The change of teleost skin commensal microbiota is associated with skin mucosal transcriptomic responses during parasitic infection by *Ichthyophthirius multifillis*

***Xiaoting Zhang*[1¶], *Liguo Ding*[1¶], *Yongyao Yu*[1], *Weiguang Kong*[1], *Yaxing Yin*[1], *Zhenyu Huang*[1], *Xuezhen Zhang*[1], *Zhen Xu*[1,2*]**

1 Department of Aquatic Animal Medicine, College of Fisheries, Huazhong Agricultural University, Wuhan, Hubei, 430070, China, **2** Laboratory for Marine Biology and Biotechnology, Qingdao National Laboratory for Marine Science and Technology, Qingdao 266071, China.

[¶]These authors contributed equally to this work.

[*]***Correspondence***:

Zhen Xu

zhenxu@mail.hzau.edu.cn

Keywords: mucosal immunity, skin, transcriptomic, microbiota, *Ichthyophthirius multifiliis*, rainbow trout (*Oncorhynchus mykiss*)

Supplementary Information

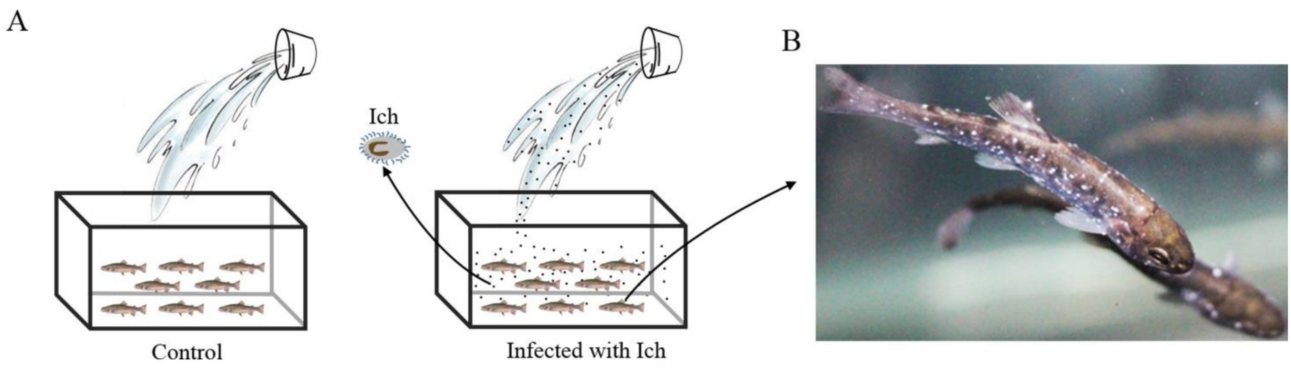

FIGURE S1. Construction of rainbow trout model for Ich infection *in vivo*. (A) Compared with control fish that were mock infected and maintained in a similar tank but without parasite, infected fish were exposed to an optimal single dose of ~ 5000 theronts per fish added directly into the aquarium. (B) The Ich parasites can be seen significantly coating on the trout skin at 7 day after infection, which indicates that the parasite mainly succeed in invading trout skin mucosal tissue.

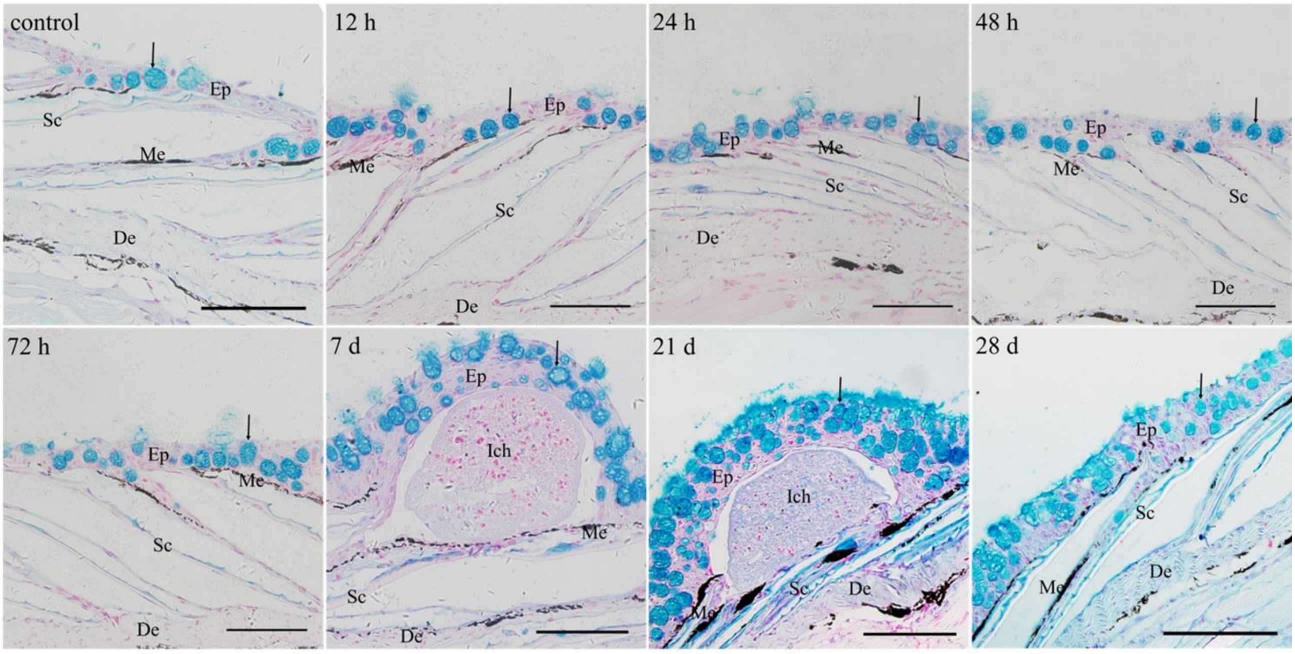

FIGURE S2. Pathological changes detected by A & B stain in skin of trout after Ich infection. The longitudinal variation of skin epidermis at different time points following the parasite infection: 12 h, 24 h, 48 h, 72 h, 7 d, 21 d, 28 d and control fish indicate the changes of mucus cells in skin epidermis (n = 6 fish per group). Skin structure is always displayed with the outside part of the epidermis (Ep) and the dermis (De). Scales (Sc) and melanophores (Me) are also indicated with black letters. The arrowhead line points to mucus cells. Scale bars, 50 μm. Data are representative of three different independent experiments.

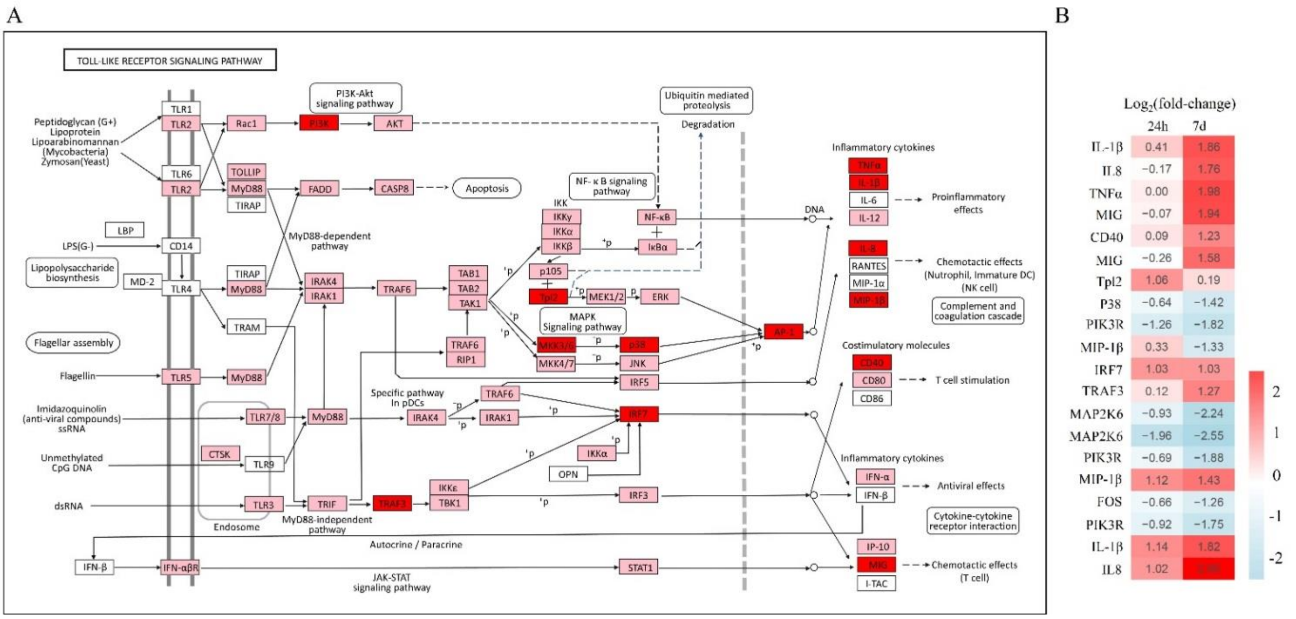

FIGURE S3. Overview of the Toll-like receptor signaling pathway in trout skin. (A) The molecules in the TLR signaling pathway identified in trout skin are shown with red and pink boxes. And the red boxes indicate DEGs in this pathway. The figure is modified from KEGG map04620. (B) Differential expression genes involved in the TLR pathway were analyzed at 24 h and 7 d post Ich infection. The color gradient represents highly up-regulated (red) to highly down-regulated (green) genes.

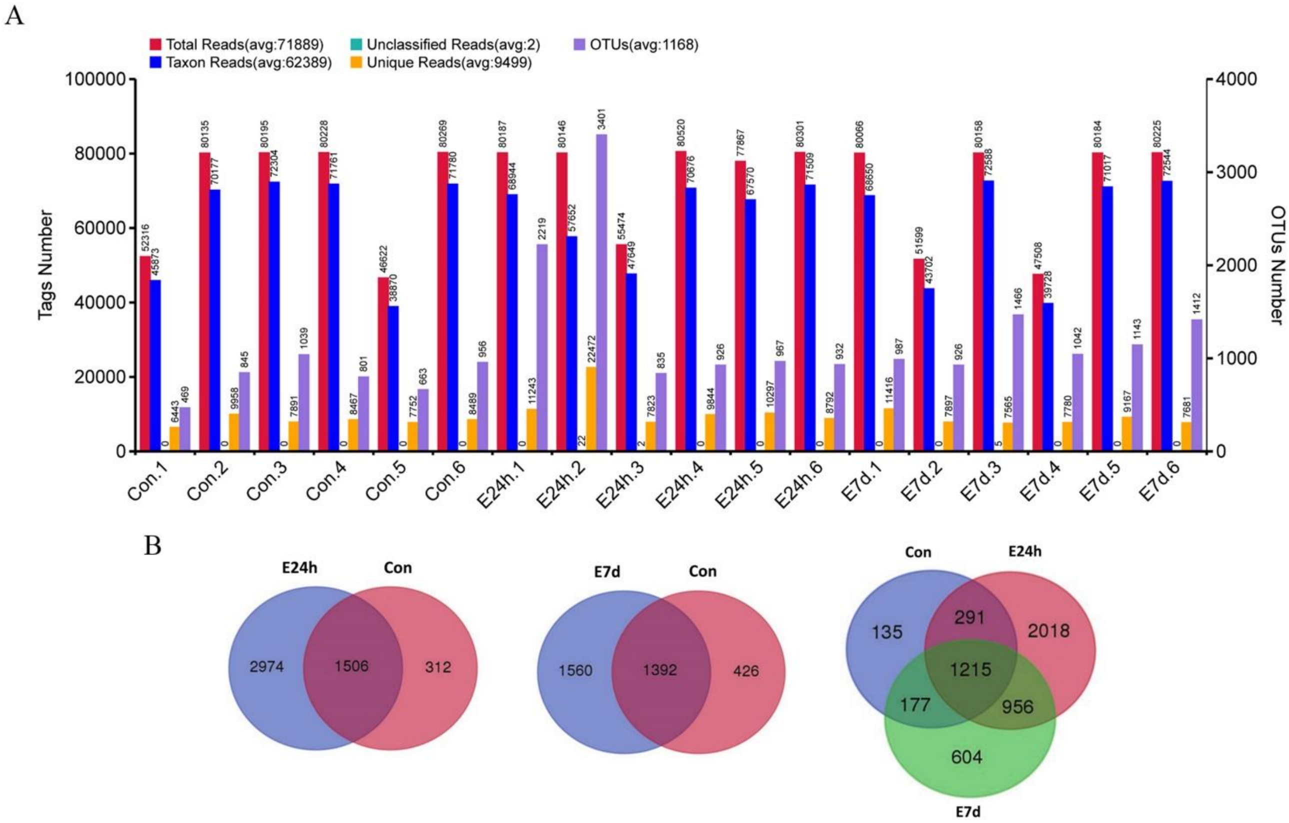

FIGURE S4. Graphical representation of the data processing. (A) After pair end merging, quality trimming and criteria filtering of raw reads obtained by Illumina run, high-quality clean reads are acquired and used to taxonomic analysis of OTUs noting. (B) Venn diagram of the OTUs indicates the overlaps and differences among infected and control groups. Red column represents the total reads; blue column represents the taxon reads; green column represents the unclassified reads; orange column represents the unique reads. All of these reads are exhibited with the left Y axis. Purple column represents the OTUs with the right Y axis.

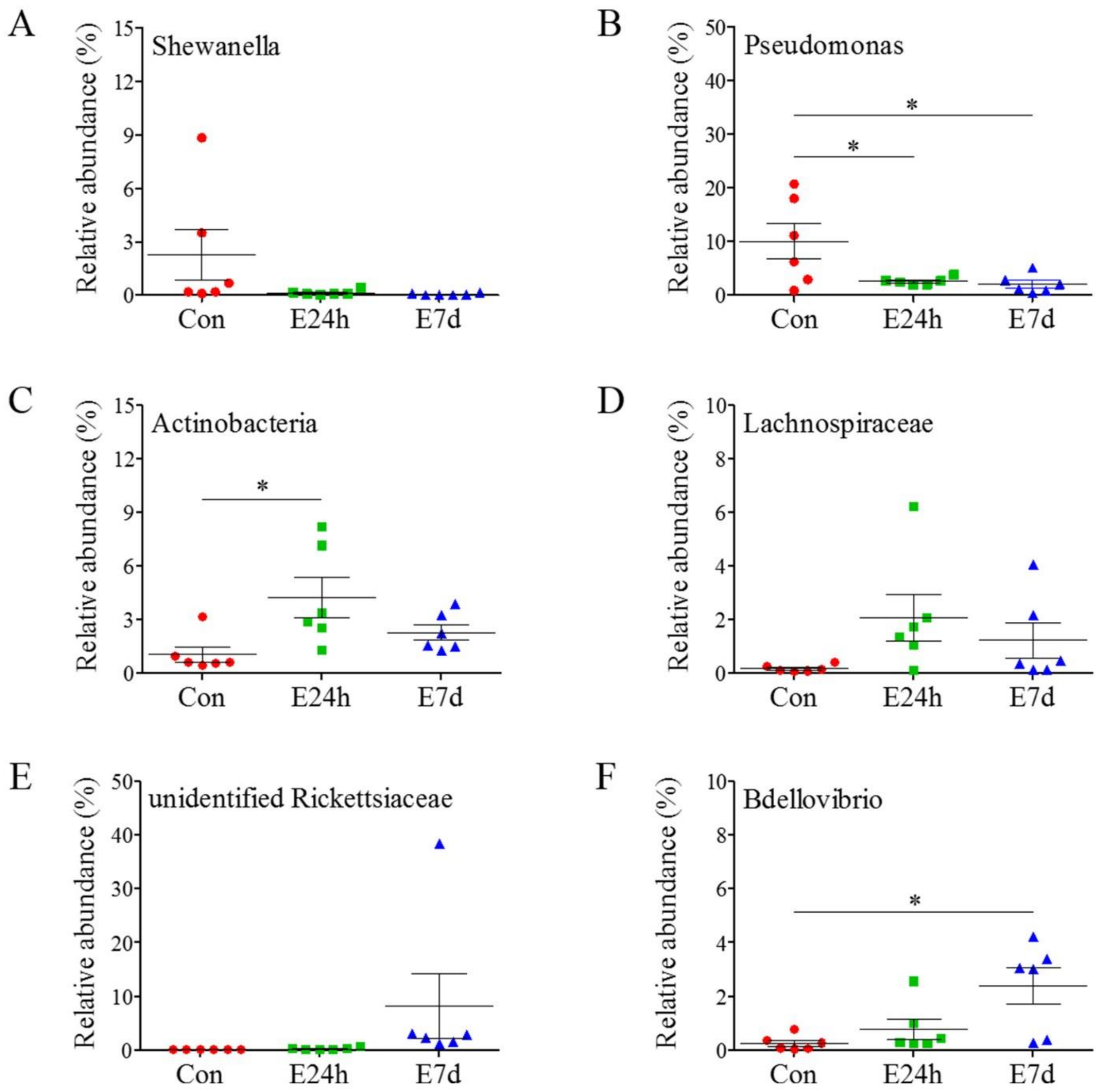

FIGURE S5. The biomarkers of trout skin microbial community following Ich-parasites invasion. Ich infection results in losses of beneficial bacteria and increased abundance of opportunistic pathogens in trout skin. Abundance of another six biomarkers at the genus level are shown as follows: (A) *Shewanella*, (B) *Pseudomonas*, (C) *Actinobacteria*, (D) *Lachnospiraceae*, (E) *Rickettsiacae* and (F) *Bdellvibrio* (mean ± s.e.m.). Con: control group. E24h: 24 hours after Ich infection. E7d: 7 days after Ich infection. * $P < 0.05$ (unpaired Student's *t*-test).

TABLE S1. Summary of Illumina sequence data.

Samples	Con_1	Con_2	Con_3	E24h_1	E24h_2	E24h_3	E7d_1	E7d_2	E7d_3
Raw Reads Number	28986309	27585276	27728049	24099925	30338480	26995404	26218210	27666563	28065683
Clean Reads Number	28354770	26868032	26576572	23563825	29600550	26438699	25657849	26900688	27467099
Clean Reads Rate (%)	97.82	97.40	97.43	97.79	97.57	97.94	97.86	97.28	97.87
Clean Reads Q20 (%)	97.56	95.05	97.62	97.12	97.33	97.15	97.33	97.55	97.48
Clean Reads Q30 (%)	93.82	88.81	93.88	92.95	93.36	92.89	93.35	93.78	93.69
GC percent (%)	51.07	50.68	51.86	51.20	50.96	50.75	50.50	50.49	50.79

TABLE S2. Reads mapping information.

Samples	Con_1	Con_2	Con_3	E24h_1	E24h_2	E24h_3	E7d_1	E7d_2	E7d_3
Total Reads	56709540	53736064	53153144	47127650	59201100	52877398	51315698	53801376	54934198
Total Map (%)	87.37	83.29	87.64	85.26	86.49	85.75	86.27	87.13	86.54
Unique Map (%)	80.99	77.32	78.94	78.29	79.96	78.83	79.34	80.17	79.26
Multi Map (%)	6.38	5.96	8.71	6.97	6.53	6.92	6.93	6.95	7.29
Splice Map (%)	38.37	35.39	40.38	35.94	37.81	36.29	36.17	36.69	36.59

TABLE S3 | KEGG enrichment of DEGs.

KEGG pathway	Description	E24h/Con		E7d/Con	
		Total no.	*P*-value	Total no.	*P*-value
ko04512	ECM-receptor interaction	35	1.53E-13	57	3.36E-13
ko04510	Focal adhesion	43	7.39E-06	78	5.05E-04
ko05410	Hypertrophic cardiomyopathy (HCM)	22	1.17E-04	49	2.72E-07
ko04610	Complement and coagulation cascades	16	2.84E-04	29	4.22E-04
ko05217	Basal cell carcinoma	7	0.516311	40	2.28E-07
ko05414	Dilated cardiomyopathy (DCM)	20	0.001659	47	9.07E-06
ko04640	Hematopoietic cell lineage	4	0.862624	32	4.75E-05
ko05144	Malaria	5	0.396126	24	7.8E-05
ko04350	TGF-beta signaling pathway	11	0.320736	42	0.00014
ko04060	Cytokine-cytokine receptor interaction	21	0.523122	79	0.00022
ko04610	Complement and coagulation cascades	16	0.000284	29	0.00042
ko05322	Systemic lupus erythematosus	3	0.904927	26	0.00077
ko05020	Prion diseases	8	0.005437	16	0.00103
ko04151	PI3K-Akt signaling pathway	53	0.002625	117	0.00288
ko05146	Amoebiasis	16	0.011587	35	0.00307
ko05150	Staphylococcus aureus infection	5	0.142011	15	0.00346
ko05412	Arrhythmogenic right ventricular cardiomyopathy	10	0.200476	35	0.00019
